# Identifying Potential Mitochondrial Proteome Signatures Associated with the Pathogenesis of Pulmonary Arterial Hypertension in the Rat Model

**DOI:** 10.1155/2022/8401924

**Published:** 2022-02-21

**Authors:** Jie Wang, Md. Nazim Uddin, Qian Li, Alidan Aierken, Ming-Yuan Li, Rui Wang, Qian-zhi Yan, Dilare Adi, Ming-Tao Gai, Yun Wu

**Affiliations:** ^1^Department of Pharmacy, First Affiliated Hospital of Xinjiang Medical University, Urumqi 830011, China; ^2^School of Basic Medicine and Clinical Pharmacy, China Pharmaceutical University, Nanjing 211198, China; ^3^Institute of Food Science and Technology, Bangladesh Council of Scientific and Industrial Research (BCSIR), Dhaka 1205, Bangladesh; ^4^Department of General Medicine, First Affiliated Hospital of Xinjiang Medical University, Urumqi 830011, China; ^5^Xinjiang Key Laboratory of Cardiovascular Disease Research, First Affiliated Hospital of Xinjiang Medical University, Urumqi 830011, China

## Abstract

Pulmonary arterial hypertension (PAH) is a severe and progressive disease that affects the heart and lungs and a global health concern that impacts individuals and society. Studies have reported that some proteins related to mitochondrial metabolic functions could play an essential role in the pathogenesis of PAH, and their specific expression and biological function are still unclear. We successfully constructed a monocrotaline- (MCT-) induced PAH rat model in the present research. Then, the label-free quantification proteomic technique was used to determine mitochondrial proteins between the PAH group (*n* = 6) and the normal group (*n* = 6). Besides, we identified 1346 mitochondrial differentially expressed proteins (DEPs) between these two groups. Gene Ontology (GO) and the Kyoto Encyclopedia of Genes and Genomes (KEGG) were used to analyze the mainly mitochondrial DEPs' biological functions and the signal pathways. Based on the protein-protein interaction (PPI) network construction and functional enrichment, we screened 19 upregulated mitochondrial genes (*Psmd1*, *Psmc4*, *Psmd13*, *Psmc2*, etc.) and 123 downregulated mitochondrial genes (*Uqcrfs1*, *Uqcrc1*, *Atp5c1*, *Atp5a1*, *Uqcrc2*, etc.) in rats with PAH. Furthermore, in an independent cohort dataset and experiments with rat lung tissue using qPCR, validation results consistently showed that 6 upregulated mitochondrial genes (*Psmd2*, *Psmc4*, *Psmc3*, *Psmc5*, *Psmd13*, and *Psmc2*) and 3 downregulated mitochondrial genes (*Lipe*, *Cat*, and *Prkce*) were significantly differentially expressed in the lung tissue of PAH rats. Using the RNAInter database, we predict potential miRNA target hub mitochondrial genes at the transcriptome level. We also identified bortezomib and carfilzomib as the potential drugs for treatment in PAH. Finally, this study provides us with a new perspective on critical biomarkers and treatment strategies in PAH.

## 1. Introduction

Pulmonary arterial hypertension (PAH) is a severe and debilitating disorder with complex pathogenesis. It is characterized by pulmonary arterioles' pathological changes, leading to progressive increases in pulmonary vascular resistance, right heart failure, and finally becoming a significant complication of premature death from other diseases [1]. PAH has been proven with a prevalence range of 15 to 52 cases per million previously and significantly the highest in females above 65 years old [2]. Moreover, in most developed countries, the 1-, 3-, and 5-year survival rates are 68%, 48%, and 34%, respectively, and the situation is even worse in underdeveloped and developing countries [3, 4]. Although several drugs can control the burden of PAH symptoms, including phosphodiesterase type 5 inhibitors, soluble guanylate cyclase stimulators, prostacyclin analogs, prostacyclin receptor agonists, and endothelin receptors antagonists, they still cannot cure or reverse the progression of PAH [5]. However, due to the unclear pathogenesis of PAH and the lack of more advanced diagnosis and treatment methods, the current aim of treatment is still to improve the quality of life in PAH patients. A retrospective study found that PAH-related hospitalizations decreased between 2001 and 2012, but the average cost and length of hospitalization with PAH increased, and the hospital mortality of PAH was not significantly decreasing [6]. Therefore, further searching for useful biomarkers and targeted therapeutic drugs is the fundamental way to cure PAH. This study is aimed at exploring potential pathogenic molecules through proteomic studies, which will provide the necessary basis for improving the treatment of PAH.

Mitochondria are semiautonomous organelles that have retained a high capacity to synthesize ATP via oxidative phosphorylation, which participates in multiple metabolisms [7]. Several studies showed that mitochondrial/metabolic dysregulation's contribution was essential in PAH. Some essential proteins, such as the redox-sensitive nuclear factor erythroid 2-related factor 2 (Nrf2), the AMP-activated protein kinase (AMPK), uncoupling protein 2 (UCP2), Nogo-B, dynamin-related protein (DRP) 1, mitofusin-2, and sarco-/endoplasmic reticulum calcium-ATPase (SERCA), are associated with mitochondrial metabolic functions in PAH [8–14]. Thus, protecting mitochondrial structure and biogenesis is a promising therapy in PAH.

Mitochondrial proteomics provides essential data for studying mitochondrial dysfunction, disease diagnostic markers, and finding target drugs. The mitochondrial proteome study can intuitively see the mitochondrial protein changes after therapy in some diseases [15–17]. High-throughput LC-MS/MS technologies have developed significantly over the last ten years. Label-free quantification proteomics is considered the most straightforward and most economical approach for studying diabetes, acute myocardial infarction, cancer, etc. [18–20]. Determining the critical protein changes in mitochondria responsible for vascular remodeling in PAH is currently essential for the PAH research field.

This study successfully constructed a monocrotaline- (MCT-) induced PAH rat model, as previously reported [21]. We identified the mitochondrial proteome using label-free quantification proteomic techniques. Then, we screened the mitochondrial DEPs between the PAH and the control groups. Functional pathway enrichment and protein-protein interaction (PPI) network analysis demonstrated that the mitochondrial DEPs involved several PAH-related biological functions and signaling pathways. Finally, we identified and validated the hub proteins and predicted the potential miRNA target to the top 20 hub proteins. This research reported a comparative proteomic analysis to investigate potential mitochondrial biomarkers, which could provide a new perspective for the targeted treatment of PAH.

## 2. Materials and Methods

### 2.1. Rat Grouping and Establishment of Animal Models

All the experiments were performed with the approval of the animal ethics committee of the First Affiliated Hospital of Xinjiang Medical University under the Care and Use of Laboratory Animals Guidelines of the National Institutes of Health (permit number: IACUC20190416-01). To ensure that the interests of the animals are fully considered during the research process, to minimize stress, pain, and injury to the animals, and to respect animal life, we choose the least painful method to dispose of all experimental animals. Twelve male Wistar rats weighing 250-280 g were randomly divided into two groups: the control group and the PAH group. Rats in the PAH group were induced intraperitoneal injection of MCT (Sigma-Aldrich; 60 mg/kg) once on the first day of the experiment. Rats in the control group received the same volume of saline. All rats in each group were continuously feeding for four weeks.

The hemodynamic indexes were measured by the right heart catheter method. According to the observation time point, after the experimental rats were anesthetized with 1% pentobarbital sodium (45 mg/kg) intraperitoneally, the right external jugular vein was isolated, a polystyrene tube was inserted, the right atrium and right ventricle were accessed, and the mean right ventricular pressure (mRVP) was measured and recorded with the BL-420F bioinformatics acquisition system. The subcutaneous fascial and muscular layers were freed by blunt separation, the trachea was released, an inverted T-shaped cut was made, and a rat tracheal tube was inserted. The animal was connected to a ventilator, the rib cage was cut, the thoracic cavity was opened, the pulmonary artery was intubated, and the BL-420F biofunctional information acquisition system was connected. The mean pulmonary artery pressure (mPAP) was measured and recorded.

After determining hemodynamic indexes, the experimental rats were administered intraperitoneally with 1% pentobarbital (100 mg/kg), and all rats were sacrificed using cervical dislocation. The lung samples from those rats were removed and rapidly washed in 0.9% precooled saline. Then, the lung tissues fixed in 10% formaldehyde solution were taken transversely from the right pulmonary hilum for routine paraffin filming, and the hematoxylin and eosin (H&E) staining was performed.

### 2.2. Mitochondrial Protein Extracted from Lung Tissue in Rats

Subsequently, the mitochondrial protein was extracted from fresh lung tissues. According to the manufacturer's instructions, mitochondria isolation was performed using a mitochondria isolation kit (Thermo Scientific, Rockford, IL, USA). The procession of mitochondrial proteins isolated by reagent-based method and described as follows: (1) 50-200 mg fresh samples from lung tissues were washed twice with 2-4 mL of PBS and then carefully remove and discard the PBS wash. (2) Cut tissue into small pieces and add 800 *μ*L of PBS, tissue disruption by 3-5 Dounce strokes on ice with a precooled Dounce tissue grinder to obtain a homogeneous suspension, and then centrifuge tissue at 1000 × *g* for 3 minutes at 4°C and discard the supernatant. (3) Add protease inhibitors in Reagent A before use, and suspend the pellet in 1 mL of BSA/Reagent A solution (4 mg/mL) and then vortex at medium speed for 5 seconds and incubate tube on ice for precisely 2 minutes. (4) Add 10 *μ*L of Reagent B and then vortex at maximum speed for 5 seconds and incubate tube on ice for 5 minutes. (5) Add 800 *μ*L of Reagent C (add protease inhibitors before use), and invert tube several times to mix. (6) Centrifuge tube at 700 × *g* for 10 minutes at 4°C and then discard pellet and transfer supernatant to a new 2 mL tube. (7) Centrifuge supernatant at 3000 × *g* for 15 minutes at 4°C and then remove the supernatant (containing cytosolic fraction) from the mitochondrial pellet. (8) Add 500 *μ*L of wash buffer to the mitochondrial pellet and perform a surface wash of the pellet and then centrifuge at 12,000 × *g* for 5 minutes and discard the supernatant. Finally, more than 50% of the organelle (e.g., lysosomes and peroxisomes) contamination was removed; the relatively high-purity mitochondrial pellet was collected and maintained on ice before downstream processing. Furthermore, the mitochondrial protein was extracted using the Mammalian Tissue & Cells Protein Extraction Kit (Bangfei Bioscience Co., Ltd., Beijing, China), and all of the chemicals used for protein extraction digestion were of analytical grade.

### 2.3. Quality Control and Digestion of Extracted Mitochondrial Protein

Protein purity and concentration were measured using a Bradford assay kit (Dingguo Biology, Guangzhou, China). Create the calibration curve using the kit manufacturer's instructions, and calculate the concentration of the configured protein solution, and then, the quantitative detection of the proteins was tested 2 replicates for each sample at 595 nm in an enzyme-labeled instrument (BioTek, H4MFPTAD). Besides, the protein samples were further separated by Coomassie brilliant blue R-250 staining following SDS-PAGE for visualization. The protein electrophoresis band of the test sample is clear, complete, and homogeneous, and the protein not degraded is considered to be qualified for quality control. The protein digestion was performed primarily following the Filter Aided Sample Preparation (FASP) protocol 1 [22]. Approximately 0.1 g of tissues was homogenized in 1 mL of an extraction buffer for 5 min using a homogenizer. The lysates were vortexed at room temperature using the maximum speed of 5 min and were then boiled at 95°C for 5 min. Then, the lysates were centrifuged at 20,000 × *g* at 25°C. Protein quantification was performed using the Bradford method. For all of the samples, three biological replicates were performed. The protein samples were reduced, alkylated with DTT and iodoacetamide (IAA), and digested with trypsin using the FASP method. All of the peptide samples were collected for mass spectrometry analysis.

### 2.4. Label-Free Quantitative Analysis and Data Processing

We used Orbitrap Fusion Lumos (Thermo Scientific) to perform label-free quantitative analysis, coupled with an Easy nLC/Ultimate 3000 System (Thermo Scientific). Analysis of peptide information was adopted by liquid chromatography- (LC-) electrospray ionization (ESI) tandem MS (MS/MS) analysis as previously described [23, 24].

For the procession of LC-MS/MS analysis, the separation of mitochondrial protein was pressure-loaded onto a fused silica capillary column packed with 3 *μ*m Dionex C18 material (RP; Phenomenex). The RP sections with 100 Å were 15 cm long, respectively, and the column was washed with buffer A (water, 0.1% formic acid) and buffer B (acetonitrile, 0.1% formic acid). After desalting, a 5 mm, 300 *μ*m C18 capture tip was placed in line with an Agilent 1100 quaternary HPLC (high-performance liquid chromatography) and analyzed using a 12-step separation.

The first step consisted of a 5 min gradient from 0% to 2% buffer B, followed by a 45 min gradient to 40% buffer B. Next, buffer B flowed by a 3 min gradient from 40% to 80% and 10 min 80% buffer B. After a 2 min buffer B gradient from 80% to 2%, approximately 100 *μ*g of tryptic peptide mixture was loaded onto the columns and separated at a flow rate of 0.5 *μ*L/min using a linear gradient. As peptides were eluted from the microcapillary column, they were electrosprayed directly into a micrOTOF-Q II mass spectrometer (BRUKER Scientific) with the application of a distal 180°C source temperature. The mass spectrometer was operated in the MS/MS (auto) mode. Survey MS scans were acquired in the TOF-Q II with the resolution set to a value of 20,000. Each survey scan (50~2500) was followed by five data-dependent tandem mass (MS/MS) scans at 2 Hz normalized scan speed.

Protein identification and label-free quantitative analysis were performed using Proteome Discover software (ver. 2.1 Thermo Fisher Scientific) in the Uniprot-Rattus_norvegicus database. Decoys for the database search were generated with the revert function. The following options were used to identify the proteins: peptide mass tolerance = ±15 ppm, MS/MS tolerance = 0.02 Da, enzyme = trypsin, missed cleavage = 2, fixed modification: carbamidomethyl (C), variable modification: oxidation (M), database pattern = decoy. The false discovery rate (FDR) for peptides and proteins was 0.01. Furthermore, this research identified the differentially expressed mitochondrial proteins that met *P* value < 0.05 and FC > 1.5 or FC < 0.677 as upregulated or downregulated mitochondrial proteins.

### 2.5. Functional Enrichment and Signal Pathway Analysis

Gene Ontology (GO) enrichment and Kyoto Encyclopedia of Genes and Genomes (KEGG) pathway analysis were the primary bioinformatics analysis method used to annotate the proteins' biological functions of genes and proteins [25, 26]. We used the “clusterProfiler” package [27] of the R program to identify the essential biological processes in GO enrichment on up and down mitochondrial DEPs. The KEGG pathway analysis was also determined and visualized the significant pathways using the “clusterProfiler” package of the R program. *P* < 0.05 was considered statistically significant.

### 2.6. Identification of Hub Proteins Based on the Protein-Protein Interaction Network

STRING is an online database used to predict PPI, essential for recognizing cell activity's molecular mechanisms in disease progression. So, we used the STRING database [28] to construct PPI networks based on up and down mitochondrial DEPs separately, and the cutoff score was selected as 0.8 to make the result with high confidence and then visualized in Cytoscape (version 3.8.0) software. Continuously, we have enriched biological functions of the up and down mitochondrial DEPs in the STRING database. Then, we manually identified the mitochondrial-related protein enrichment results. Besides, we identified the hub up and down mitochondrial-related proteins separately by using the cytoHubba plugin [29] in Cytoscape software. The hub proteins with the contribution (degree) of more than 5 in the whole network were screened as the mitochondrial-related hub proteins.

### 2.7. Identifying and Verifying the Mitochondrial-Related Genes in an Independent Dataset

To demonstrate the RNA level's expression of the crucial proteins, we downloaded the GSE149713 dataset [30] from the GEO database as an independent group for validation. The rat lung tissue transcriptional profiles were analyzed based on the GPL14844 platform (Illumina HiSeq 2000). RNA sequencing was used to analyze the transcriptome in control (*n* = 4) and PAH module rats injected with MCT for 1, 2, 3, and 4 weeks (*n* = 12, each group with 3 repetitions). Furthermore, to align with our research condition, we selected the control and 4-week groups to identify differentially expressed genes (DEGs). The “Deseq2” package of the R program was used to determine the DEGs in the dataset. Genes with the absolute value of ∣log_2_FC | >0.5 and *P* < 0.05 were used as the cutoff criteria of DEG analysis. In addition, to identify coexpressed mitochondria-related genes in GSE149713, we performed interaction research of mitochondrial-related genes with up- and downregulated DEGs. Finally, we have defined the interacted mitochondrial-related genes as hub genes and verified the expression level of these hub genes in GSE149713.

### 2.8. Validation of Key Gene Expression in Lung Tissue of PAH Rats

We have identified essential genes that are differentially expressed in the mitochondria of PAH rat lung tissue through a comprehensive bioinformatics analysis based on proteomic assays and validation analysis in an independent cohort. To validate the consistency of the screening analysis results of these genes expressed in PAH rat lung tissues, we further collected the RNA from the rat's lung tissue samples (5 samples from PAH rats *vs.* 5 samples from normal rats) and then performed the quantitative real-time PCR to determine the expression level of the critical genes in rat's lung tissue between PAH and normal group. Rat lung tissue RNA was extracted using the Trizol solution (Invitrogen, Carlsbad, CA, USA), the primary process followed by the following steps: grinding frozen rat lung tissue with liquid nitrogen and adding precooled Trizol (frozen tissue : Trizol = 0.1 g : 1 mL); vortexing and mixing, centrifuging at 12,000*g* for 5 min at 4°C (repeated 2 times to ensure clean protein removal); adding 200 *μ*L of chloroform, mixing rapidly for 15 s, stationary for 2-3 min, and then centrifuged at 12,000*g* for 5 min; take the supernatant (approximate 600 *μ*L), add 500 *μ*L of precooled isopropanol and mix gently, put into -20°C for about 30 min and then centrifuge at 12,000*g* for 10 min; discard the supernatant, wash the precipitate with 70% or 75% ethanol (1 mL), dry on an ultraclean table for 5 min, collect the RNA, and store at -20°C after dissolving with DEPC water. Synthesis of cDNA first strand was done using reverse transcription kit (Foregene, Chengdu, China). The expression levels of critical genes were quantified using SYBR Green Master Mix (SYBRGREEN, Beijing, China). The primers used in qPCR are listed in Table 1. qRT-PCR was performed on ViiA™ 7 System software (Thermo Fisher Scientific, ABI7500, USA). The results were normalized to the expression of *Gapdh* and presented as the fold change (2^−*ΔΔ*CT^).

### 2.9. Prediction of the miRNA Target to the Hub Mitochondrial-Related Proteins

To further investigate the potential regulation of hub proteins at the transcriptome level, we used the RNAInter database for predicting the miRNA target to the hub mitochondrial-related proteins [31]. This database has established an integrated, experimentally, verified, and computationally predicted knowledge base of RNA-related interactions, manually collating literature and another 35 resources under a common framework. It could provide a new understanding of the interaction between key mitochondrial-related proteins and RNAs.

### 2.10. Identification of Food and Drug Administration- (FDA-) Approved Drug-Hub Mitochondrial-Related Gene Interaction

We identified the potential candidate drugs that target the hub mitochondrial-related genes by using the DGIdb [32]. DGIdb collects drug-gene interaction data from 30 disparate sources, including ChEMBL, DrugBank, Ensembl, NCBI Entrez, PharmGKB, PubChem, Clinical Trial Databases, and literature in NCBI PubMed. The drug-gene interactions supported by at least one database and PubMed reference were identified. We selected the only drugs that the FDA has approved.

## 3. Results

### 3.1. The Successful Construction of the Rat PAH Model

**Figure 1:**
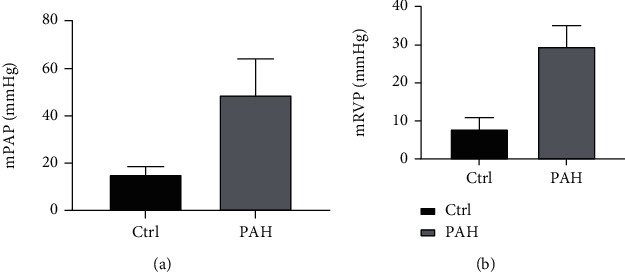
The result of hemodynamic indicators between the control and PAH groups. (a) Rats in the PAH group had a higher mPAP value than rats in the control group. (b) Rats in the PAH group had a higher mRVP value than rats in the control group.

**Figure 2:**
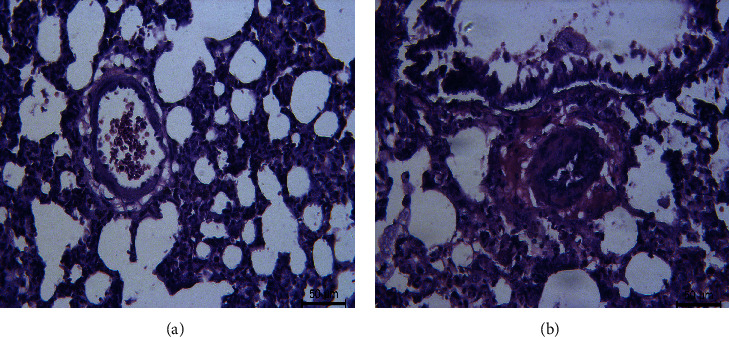
The H&E staining image of the lung tissue sample from rats in different groups. (a) The normal tube wall is normal. (b) The film thickness in the model group was thickened.

To certify the successful construction of the rat PAH model, we evaluated the hemodynamic indexes. The mPAP and mRVP values of rats in the PAH group were higher than those of the control group (*P* < 0.001). The results of hemodynamic indicators of rats between control and PAH groups are shown in Figure 1. Furthermore, H&E staining images of lung tissue samples from rats in different groups indicated that the animal model was successfully constructed (Figure 2).

### 3.2. Quality Control of Extracted Proteins

**Figure 3:**
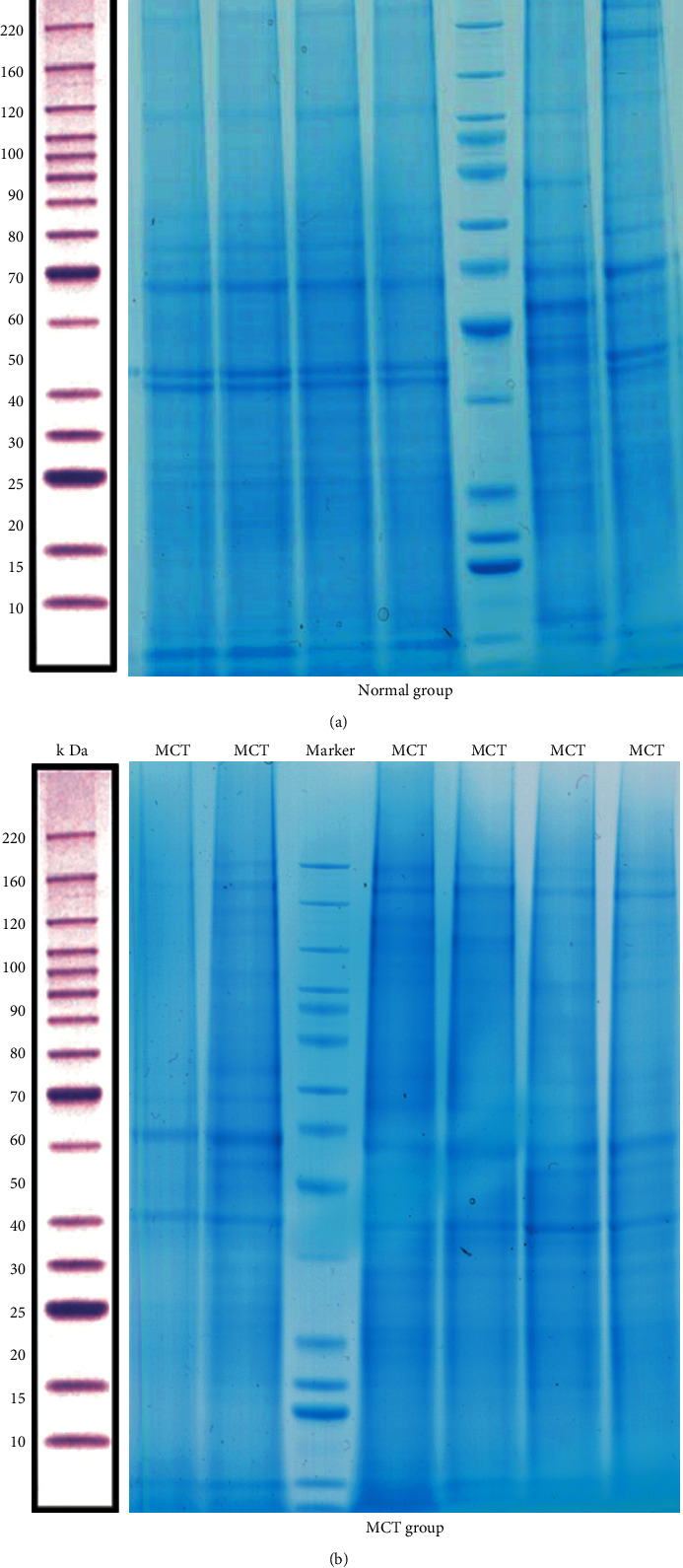
The total proteins are separated by SDS-PAG electrophoresis. (a) The protein in the normal group was visualized by Coomassie brilliant blue staining. (b) The protein in the MCT group was visualization by Coomassie brilliant blue staining.

In our study, we quantified the extracted protein samples by enzyme standardization and visualized them by gel electrophoresis, respectively, to ensure that the extracted proteins could meet the requirements of proteomic assays. The protein quantification is showed in Table 2. SDS-PAG electrophoresis showed that the total proteins were separated in the molecular weight range of 15-220 kDa without degradation, and the results of Coomassie brilliant blue staining results showed similarity in the protein pattern of the normal group. In contrast, the overall protein pattern of the model group was different (Figure 3). These results indicated that the extracted proteins could meet the requirements for subsequent proteomic analysis.

### 3.3. Characterization of Quantitative Proteomics and Differential Expression Mitochondrial Proteins

**Figure 4:**
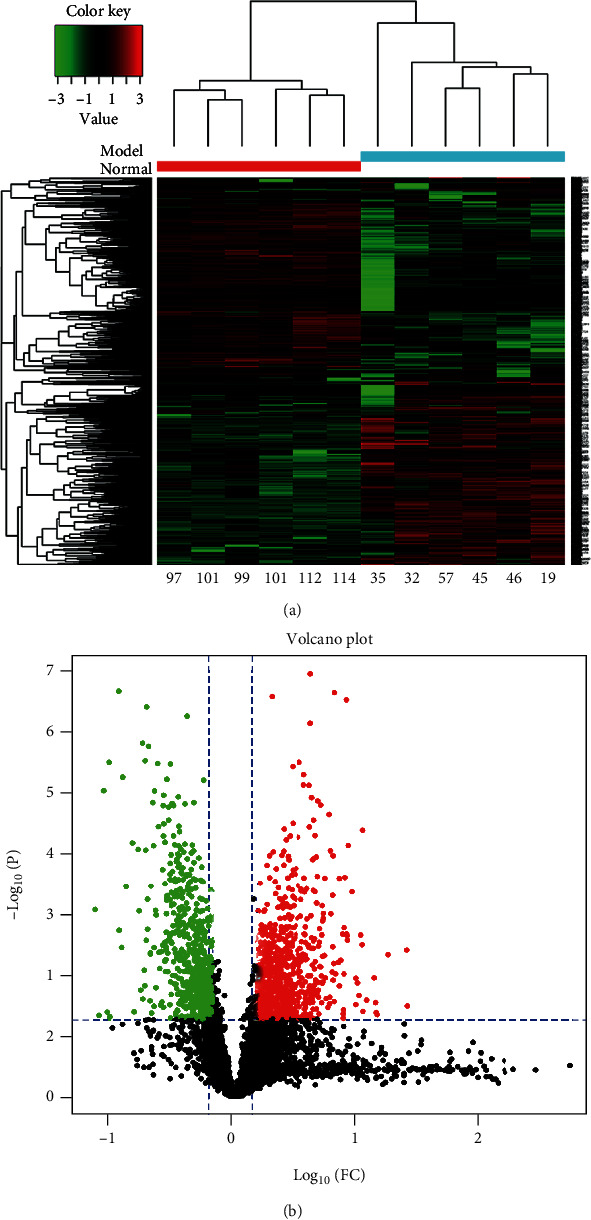
Significant mitochondrial DEPs between the PAH group and the control group. (a) The heat map for unsupervised hierarchical clustering analysis of the mitochondrial DEPs in the PAH group relative to the control group. On the *x*-axis, light blue represented the PAH group samples, and red represented the control samples. (b) The volcano plot of mitochondrial DEPs, where red dots represented the upregulated proteins and green dots represented the downregulated proteins. Mitochondrial DEPs have differentially expressed mitochondrial proteins between the PAH and normal groups.

Totally, we have obtained 1,357,614 MS/MS spectra and matched them to 38,560 distinct peptides at 0.05 *P* value cutoff, corresponding to 5663 detected protein groups (Supplementary Table S1). After data preprocessing, we studied the changes in mitochondrial protein expression patterns to identify the significant mitochondrial DEPs between the PAH and normal groups. We screened 1346 mitochondrial DEPs between these two groups (Supplementary Table S2), of which 737 proteins were upregulated and 609 proteins were downregulated. Furthermore, hierarchical clustering analysis of these DEPs was performed according to the protein expression level (Figure 4(a)). Simultaneously, the volcano plot showed the *P* value and FC distribution of these mitochondrial DEPs (Figure 4(b)).

### 3.4. Differentially Expressed Proteins Reveal Different Biological Functions and Pathways

**Figure 5:**
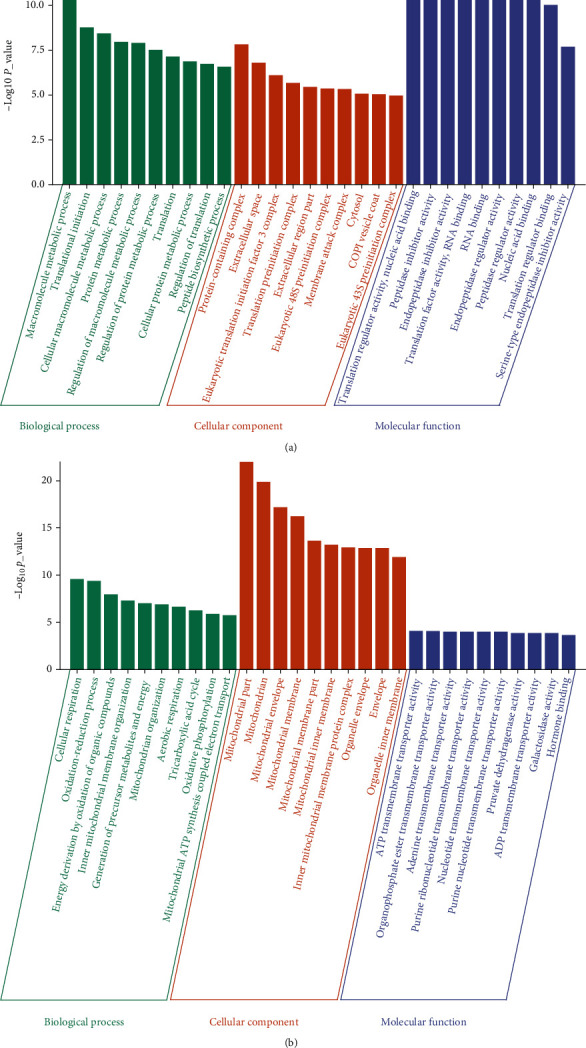
The GO functional enrichment results of up- and downregulated mitochondrial DEPs in PAH rats. (a) The top 10 BP, CC, and MF enrichment results of upregulated mitochondrial DEPs. (b) The top 10 BP, CC, and MF enrichment results of downregulated mitochondrial DEPs.

To investigate the biological functions of up- and downregulated mitochondrial DEPs in PAH rats, GO functional enrichment and KEGG pathway analyses were performed to analyze the proteins that we have identified as mitochondrial DEPs. GO functional enrichments were annotated using biological process (BP), cellular component (CC), and molecular function (MF). The BP analysis results showed that the upregulated mitochondrial DEPs were mainly involved in the macromolecule metabolic process, translation process, and peptide biosynthetic process. The CC analysis result indicated that the upregulated mitochondrial DEPs were primarily enriched in the protein-containing complex, extracellular space, translation preinitiation complex, and cytosol. The MF analysis result showed that the upregulated mitochondrial DEPs were mainly involved in the translation regulator activity, nucleic acid binding, translation factor activity, and RNA binding (Figure 5(a)).

Furthermore, the BP analysis results showed that the downregulated mitochondrial DEPs were mainly involved in the cellular respiration, oxidation-reduction process, generation of precursor metabolites and energy, inner mitochondrial membrane organization, mitochondrion organization, and mitochondrial ATP synthesis-coupled electron transport. The CC analysis results suggest that the downregulated mitochondrial DEPs were mainly enriched in mitochondrial part, mitochondrion, mitochondrial envelope, mitochondrial membrane part, mitochondrial inner membrane, and inner mitochondrial membrane protein complex. The MF analysis results indicated that the downregulated mitochondrial DEPs were involved in ATP transmembrane transporter activity, organophosphate transmembrane transporter activity, and nucleotide transmembrane transporter activity (Figure 5(b)).

**Figure 6:**
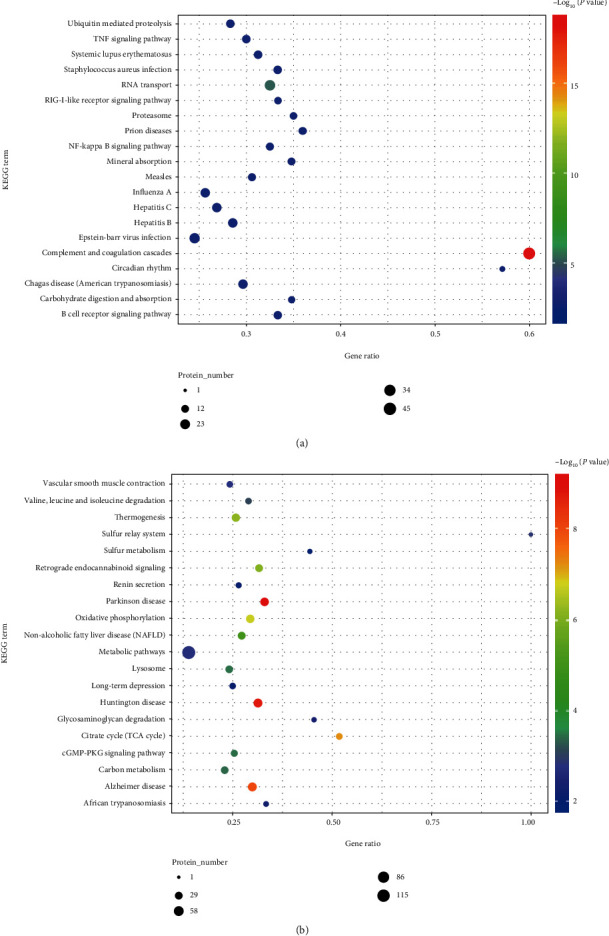
The KEGG pathway annotation results of up- and downregulated mitochondrial DEPs in PAH rats. (a) The KEGG pathway analysis results of upregulated mitochondrial DEPs. (b) The results of the KEGG pathway analysis of downregulated mitochondrial DEPs.

Moreover, the pathway enrichment analysis results showed that the upregulated mitochondrial DEPs were closely associated with aging and inflammatory processes, such as ubiquitin-mediated proteolysis, TNF signaling pathway, RIG-I-like receptor signaling pathway, and NF–kappa B signaling pathway (Figure 6(a)). The downregulated mitochondrial DEPs were mainly involved in vascular smooth muscle contraction, oxidative phosphorylation, metabolic pathways, and the cGMP−PKG signaling pathway (Figure 6(b)). Altogether, these results suggest that mitochondrial proteins may regulate the physiological functional activities of PAH through multiple biological processes.

### 3.5. Identification of Mitochondrial-Related Proteins Based on the Construction of the PPI Network

**Figure 7:**
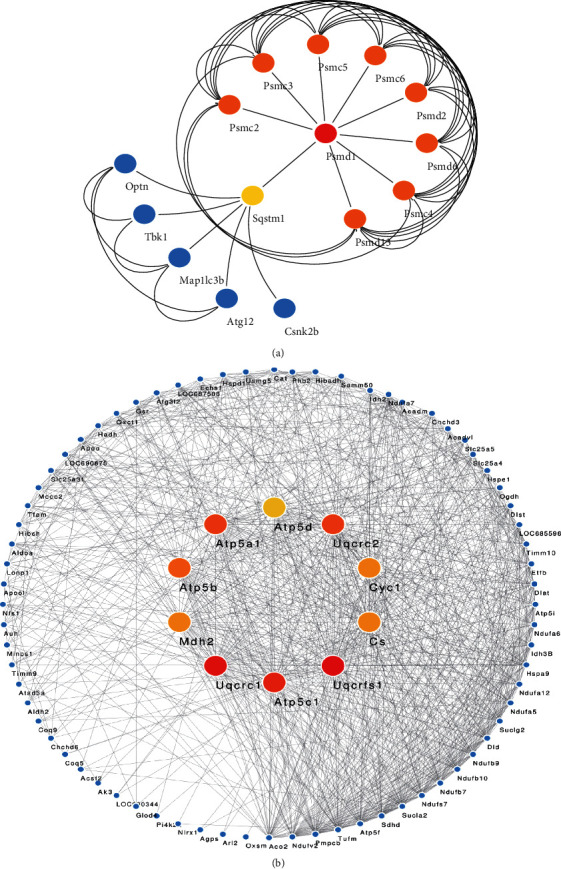
The subnetworks of mitochondrial-related genes identified from the PPI network. (a) The top 10 proteins in subnetworks of upregulated mitochondrial-related genes. (b) The top 10 proteins in subnetworks of downregulated mitochondrial-related genes. The dark nodes (red) show more significance in connectivity, the stronger the ability to regulate function in the network.

To investigate the interactions between mitochondrial DEPs, we further constructed a PPI network based on the STRING database. The protein-protein interactions of up- and downregulated proteins were visualized using the Cytoscape software (version 3.8.0) and shown in Supplementary Figures S1 and S2. To further identify the mitochondrial-related DEPs, we have analyzed the functional enrichment results of the PPI network and then got 19 upregulated proteins and 123 downregulated proteins significantly associated with mitochondria (Supplementary Tables S3 and S4). Besides, the top 10 mitochondrial-related DEPs were analyzed and visualized by the cytoHubba plugin in Cytoscape software (version 3.8.0) (Figure 7).

### 3.6. Identifying and Verifying the Hub Mitochondrial-Related Genes in an Independent Dataset

**Figure 8:**
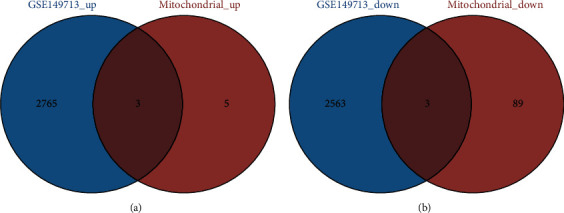
The Venn plot of mitochondrial-related genes interacted with DEGs in GSE149713. (a) The interaction results showed that 6 common upregulated genes were identified between GSE149713 and mitochondrial up genes. (b) The interaction results showed that 3 common downregulated genes were identified between GSE149713 and mitochondrial up genes.

**Figure 9:**
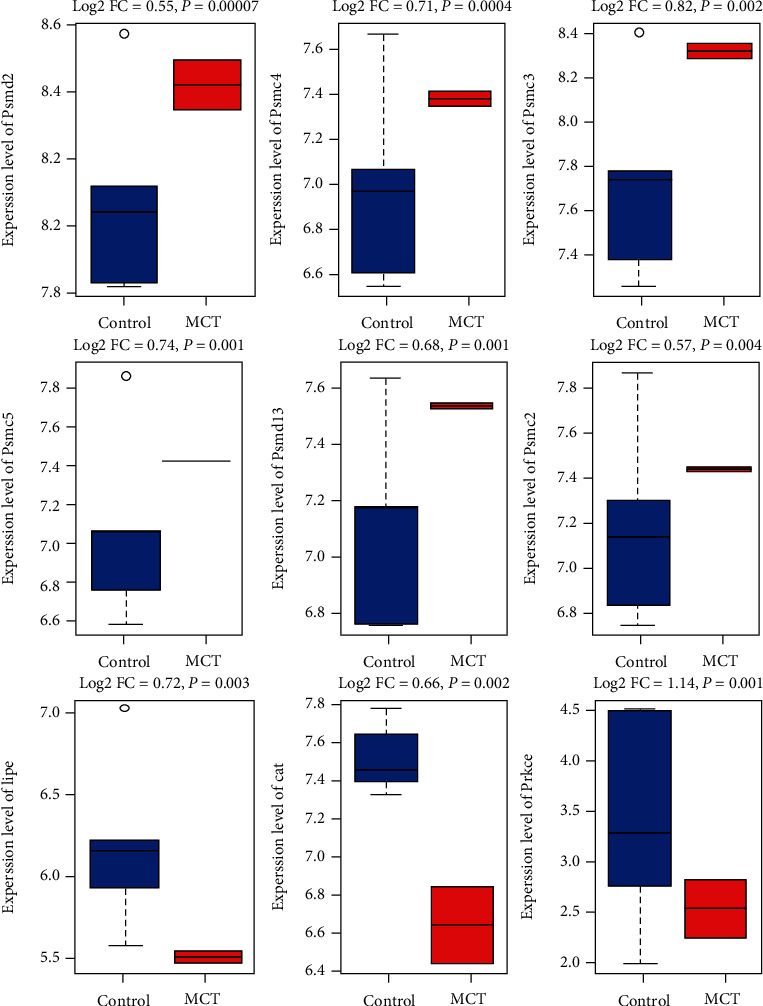
Validation of hub mitochondrial-related gene expression level in the GSE149713 dataset. The results show that 9 hub genes are significantly differentially expressed in PAH rats.

In this study, we have identified essential genes which are significantly related to the mitochondria in PAH rats. However, to further validate the specific expression of these mitochondria-related genes in external samples, we also used another independent cohort from the GEO149713 dataset to verify the hub genes. Notably, the interaction results showed that 6 common upregulated genes (*Psmd2*, *Psmc4*, *Psmc3*, *Psmc5*, *Psmd13*, and *Psmc2*) and 3 common downregulated genes (*Lipe*, *Cat*, and *Prkce*) were included in two datasets (Figure 8), and these 9 genes significantly differentially expressed in GSE149713 as hub mitochondrial-related genes in PAH rats (Figure 9). It indicates that the mitochondrial proteomes might contribute to the PAH pathogenesis.

### 3.7. Validation of Key Mitochondrial Gene Expression in Lung Tissue of PAH Rats

**Figure 10:**
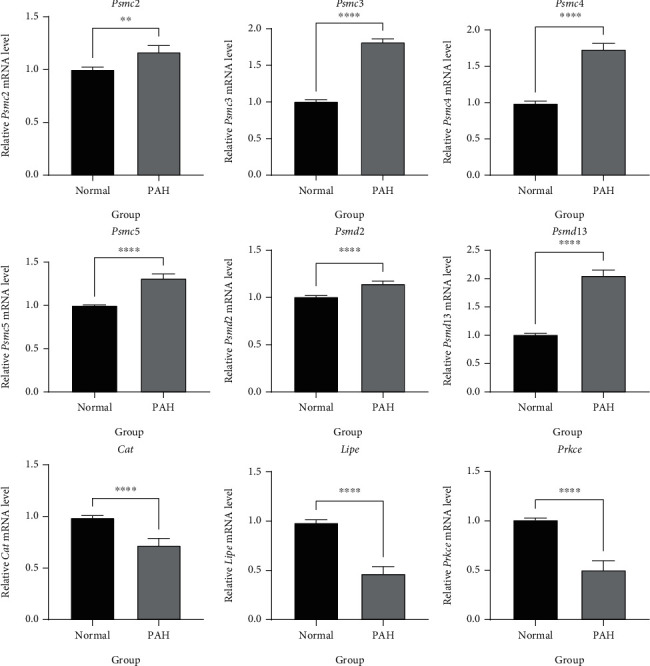
The differential expression of critical genes in the lung tissue between rats with PAH and normal. The result showed that six upregulated essential genes are significantly highly expressed in the lung tissues of rats with PAH, and three downregulated key genes are substantially lower expressed in the lung tissues of rats with PAH. ^∗∗^*P* < 0.001; ^∗∗∗∗^*P* < 0.0001.

To verify the specific expression of critical genes in the lung tissue of PAH rats, we further determined the expression of nine identified essential genes (six upregulated genes *Psmd2*, *Psmc4*, *Psmc3*, *Psmc5*, *Psmd13*, and *Psmc2*; three downregulated genes *Lipe*, *Cat*, and *Prkce*) in the lung tissue between PAH rats and normal rats using qPCR. The results showed that these key genes were aberrantly expressed in rat lung tissues and were consistent with the results of independent cohort validation (Figure 10), suggesting that all these nine key genes may play an essential biological function in the pathogenesis of PAH.

### 3.8. Prediction of the miRNA Target to the Hub Mitochondrial Genes

**Figure 11:**
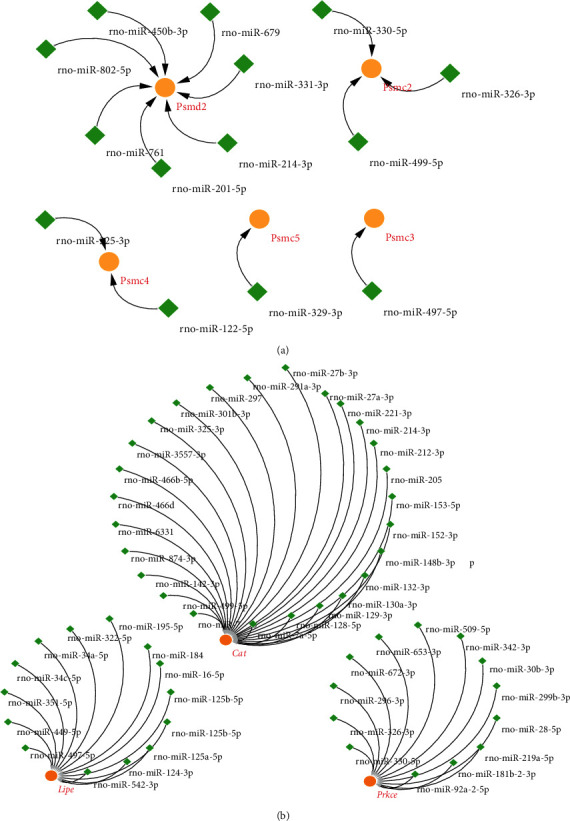
The regulation network of mitochondrial-related genes and predict miRNAs. (a) The regulation networks of common up mitochondrial genes and the potential miRNAs, orange circles represent the common up genes, and green rhombus represents the potential miRNAs. (b) The regulation networks of common down mitochondrial genes and the potential miRNAs, orange circles represent the common down genes, and green rhombus represents the potential miRNAs.

To explore the potential regulation of hub proteins at the transcriptome level, we predicted miRNAs targeting the common up/down mitochondrial-related genes. We obtained 32 miRNAs targeted to the commonly upregulated genes and 53 miRNAs targeted to the common downregulated genes (Figure 11). Interestingly, the prediction result showed that the down mitochondrial-related genes as *Cat* with the most regulations could be targeted and regulated by 26 miRNAs.

### 3.9. Identification of Potential Drugs Targeting Hub Mitochondrial Genes

We screened night overlap hub mitochondrial genes for drug-gene interactions using DGIdb, and we identified FDA-approved drugs that potentially target the protein products of four 6 hub genes (*Psmd2*, *Psmc4*, *Psmc3*, *Psmc5*, *Psmd13*, and *Psmc2*). Furthermore, the drugs could act an inhibitor role in PAH, and the proteasome inhibitors such as bortezomib and carfilzomib could have potential effects for reversing pulmonary arterial hypertension (Table 3). However, there is limited research on testing these new drugs to treat PAH as far as we know. Our data suggest that these drugs have strong potential applications and positive effects on the treatment of PAH.

## 4. Discussion

Pulmonary arterial hypertension is a branch of pulmonary hypertension, and PAH has complicated pathogenesis. Some studies have shown that PAH's proteomic analysis is mainly dedicated to new disease biomarkers and treatment targets in recent years. Therefore, identify new biological markers and target signals to explore the specific mechanisms that can reverse pulmonary vascular remodeling. The present study performed mitochondria proteomic analysis based on label-free quantification proteomic technique to comprehensively investigate potential mitochondrial biomarkers in PAH [33]. Label-free is a mass spectrometric analysis of enzymatically digested peptides by liquid mass spectrometry, eliminating the need for expensive stable isotope labels as internal standards [34]. This technology's advantages are no labeling process, simple operation, low cost, short experimental cycle time, and the ability to quantify total protein differences in any sample without restriction on sample type, making it suitable for quantitative comparisons of large sample sizes [35]. Thus, this technical approach has been applied to proteomic-related studies of many types and various organisms. In recent years, studies have been conducted using label-free protein identification techniques to analyze differential proteins in different rat disease models [36]. Moreover, it has provided technical support in analyzing mitochondrial proteins in studies such as rat myocardial injury, liver injury due to mitochondrial dysfunction, renal tubular acidosis, and central nervous system diseases [37, 38]. However, the use of the label-free technique for proteomic studies in rat models of pulmonary arterial hypertension is still minimal, primarily through the identification and discovery of the mitochondrial proteome, which can provide new insights to reveal the mechanism of PAH development further, and also, this is one of the important innovations of our study.

Mitochondria are essential organelles of eukaryotic cells, which are the center of the organism's energy metabolism and involved in various critical cytopathological processes. Mitochondrial proteins are involved in organisms' physiological and pathological processes, such as electron transport and ATP synthesis, tricarboxylic acid cycle, fatty acid oxidation, and amino acid degradation [39]. Alterations in mitochondrial protein structure and function are associated with many diseases, such as degenerative diseases, heart disease, aging, and cancer [40]. The use of proteomic research techniques to investigate the trends and interrelationships of these proteins in physiological and pathological states as a whole can provide new and robust support for the exploration of mitochondrial mechanisms of action. It is worth noting that our present study has found that many mitochondrial-related proteins in MCT-induced PAH rats exhibited expression dysregulation, including 737 upregulated and 609 downregulated proteins (Supplementary Table S2). Some up- and downregulated proteins are significantly located in the mitochondrion and play an essential role in PAH development. Hk3 is one of the top upregulated DEPs. It is considered a necessary protein with catalytic hexose phosphorylation, which can participate in carbohydrate metabolism in the hexose metabolic pathway [41]. Ndufa7, a significantly low-expressed mitochondrial-associated protein identified in the results of proteomic analysis, is an accessory subunit of the mitochondrial membrane respiratory chain NADH dehydrogenase, which plays an essential role in transferring electrons from NADH to the respiratory chain [42]. Pla2g2a, a member of the secreted phospholipase family, is another significant downregulated protein in our findings. It has been found to play an essential role in inflammation and atherosclerosis. In recent years, Pla2g2a was found to affect the process of energy metabolism and insulin sensitivity in mice by activating mitochondrial uncoupling in mouse brown adipose tissue (BAT) [43]. These results suggest that mitochondrial dysfunction and metabolic abnormalities may be an important pathological process in PAH.

In addition, based on functional enrichment analysis of these dysregulated proteins, we found that the upregulated mitochondrial DEPs were closely associated with ubiquitin-mediated proteolysis, TNF signaling pathway, RIG-I-like receptor signaling pathway, and NF–*κ*B signaling pathway (Figure 6(a)). A study reports that the ubiquitin-proteasome system (UPS) can regulate many cellular processes in the posttranslational machinery, including protein degradation, gene expression, signal transduction, and apoptosis [44]. Although the exact mechanism still needs to be fully elucidated, recent researches suggest that the ubiquitin-proteasome complex may be involved in the proliferation of PASMC in PAH and may therefore represent a new target for PAH therapy [45, 46]. Furthermore, it has been found that the pathological process of PAH can lead to an increased ubiquitin-proteasome system- (UPS-) mediated muscle protein degradation and mitochondrial abnormalities, which in turn cause extensive abnormalities in skeletal and respiratory muscles of PAH patients, ultimately causing PAH-related restriction of motor function [47]. Previous studies have reported that NF-*κ*B can regulate gene expression and then be involved in many biological processes, such as inflammatory factor production, cell proliferation, effector cell survival, and differentiation, and that the NF-*κ*B signaling pathway is aberrantly activated in PAH models [48, 49]. The new anesthetic sevoflurane can downregulate the levels of p-I*κ*B, p-P65, and P65 to inhibit NF-*κ*B signaling pathway activation in PAH rat models, thereby reducing lung fibrosis and ultimately preventing PAH [50]. Current research has revealed that the members of the RIG-I-like receptor family mainly include RIG-I, MDA5, and LGP2, of which RIG-I is the most studied pattern recognition receptor in clinical practice and plays an essential role in innate immunity [51]. Furthermore, it was demonstrated that the RIG-I-like receptor recruits MAVS (mitochondrial antiviral signaling protein) under ubiquitination modification by TRIM25 ubiquitin ligase, which in turn assembles with TRAF family members related to nuclear factor *κ*B (NF-*κ*B) activator (TANK), TNFR1-associated death transmission domain protein (TRADD) together to form “signalosome.” In turn, it leads to phosphorylation and nuclear translocation of interferon regulatory factor 3 (IRF3) by TANK-binding kinase 1 (TBK1) and IKK*ε*, ultimately activating NF-*κ*B to induce type I interferon (IFN) and proinflammatory cytokines [52]. It is worth considering that, as two synergistic degradation systems, ubiquitin-proteasome and autophagy coexist in mammalian cells, although their activities are not interdependent; recent studies have shown that there are connections and crosstalk between the two systems and that the two pathways can synergistically complement each other to maintain cellular homeostasis for closely monitoring and dealing with toxicity caused by protein misfolding [53, 54]. Furthermore, despite their different modes of action and requirements for substrate recognition, both degradation pathways can be coactivated to degrade misfolded proteins [55]. Additionally, new studies involving the role of noncoding RNAs and their complex networks in the regulation and coordination of autophagy and ubiquitin-proteasome may provide new insights into the role of mitochondrial dysfunction in the development of PAH [56].

Similarly, based on analysis in the STRING database, we also found that many dysregulated mitochondrial DEPs participate in mitochondrial autophagy (Supplementary Table S3). Currently, it has been demonstrated that PINK1-Parkin signaling is one of the classical pathways mediating mitochondrial autophagy, as well as an important pathophysiological feature of pulmonary vascular remodeling as well as PAH [57, 58]. It is worth noting that mitochondrial-related proteins such as Optn and Tbk1 identified in our study were shown to be critical proteins involved in PINK1-Parkin pathway-mediated mitochondrial autophagy [59, 60]. More specifically, when under stress conditions, the adapter protein Optn recognizes the phosphorylated polyubiquitin chains on mitochondrial proteins and initiates autophagosome formation by binding to Lc3 [61]. Besides, Tbk1 phosphorylates Optn, thereby enhancing its binding affinity to the ubiquitin chains [62]. Therefore, the Optn-Tbk1 complex could promote mitochondrial clearance by establishing a feed-forward mechanism. However, the role of mitochondrial autophagy in the pathogenesis of PAH is still not clear, and the research focus on mitochondrial autophagy-related proteins will provide novel insight into the pathogenesis of PAH.

In this study, another important finding has shown that the hub mitochondrial-related proteins such as *Psmd2*, *Psmc4*, *Psmc3*, *Psmc5*, *Psmd13*, and *Psmc2* are upregulated in PAH rat lung tissue, and there are 32 miRNAs targeted to these hub genes for regulating the occurrence of rats with PAH. The ubiquitin-proteasome system degrades ubiquitin-modified proteins to maintain proteostasis and control signaling. Ubiquitin-proteasome pathway-mediated protein degradation is a complex and sophisticated process. This highly selective protein degradation pathway plays a vital role in the cell cycle, gene transcription and expression, and antigen presentation [63]. However, ubiquitin-proteasome pathway formation's molecular mechanism and function are not well understood so far. It has been reported that 26S proteasome non-ATPase regulatory subunit 2 and 13 (*Psmd2* and *Psmd13*) are the components of 26S proteasome and capable of forming multiple protein complexes involved in the degradation of ATP-dependent ubiquitinated proteins, which play a crucial role in maintaining protein homeostasis by removing misfolded or damaged proteins that may impair cellular function and by eliminating proteins that are no longer required for function [64]. Thus, these genes are involved in cellular processes and play an essential role in cell cycle progression, apoptosis, or DNA damage repair. Studies have reported that Psmd2 could regulate breast cancer cell proliferation and cell cycle progression by regulating p21 and p27 proteasome degradation [65]. *Psmd2* regulates the expression of neo-lipid synthesis-related genes through p38-JNK and AKT signaling, thereby increasing the proliferation of HepG2 cells by promoting cellular lipid droplets, and highly expressed *Psmd2* is significantly associated with poor prognosis in hepatocellular carcinoma [66]. It was found that high expression of *Psmd13* may be closely related to the development of neuroinflammatory diseases and that silencing of *Psmd13* regulates ubiquitin-proteasome system-mediated neuroinflammation by downregulating the degradation of I*κ*B*α* and activation of NF-*κ*B in LPS-stimulated BV2 microglia, thereby inhibiting the production of proinflammatory mediators and ultimately having a therapeutic effect on neuroinflammatory diseases [64]. Furthermore, we have identified *Psmc2*, *Psmc3*, *Psmc4*, and *Psmc5* highly expressed in PAH rat lung tissue. These genes are components of the 26S proteasome and can form multiple protein complexes involved in the degradation of ATP-dependent ubiquitinated proteins [67]. Recently, the research found that *Psmc2* expression is upregulated in tumor tissues of p21-HBx transgenic mice, thereby enhancing the ubiquitin-proteasome and lysosomal pathways and contributing to the development of HBx-associated hepatocellular carcinoma [68]. A study based on whole-genome sequencing and a *Psmc3* knockout zebrafish model shows that mutations in the proteasome subunit *Psmc3* can lead to neurosensory syndrome, resulting in proteotoxic stress development, deafness, and cataracts [69]. In a study on the role of the ubiquitin-proteasome network in prostate cancer, the proteasome genes *Psmc4* and *Psmb5* and the E3 ubiquitin ligase NEDD4L were found to be significantly and consistently upregulated in prostate cancer cells compared to the corresponding adjacent normal prostate tissue, suggesting a pivotal role for *Psmc4* in prostate tumorigenesis [70]. Furthermore, a study on neurodegenerative diseases, *Psmc4*, was also found to be specifically distributed and accumulated in nigrostriatal dopaminergic neuronal cells of Parkinson's patients, suggesting that abnormalities in the ubiquitin-proteasome and autophagy systems may be major players in protein misfolding and aggregation in Parkinson's disease [71]. In comprehensive bioinformatics analysis studies, *Psmc5* has been identified as an essential protein involved in respiratory diseases, Parkinson's disease, and many other neurodegenerative diseases [72, 73].

Based on the current findings, we tentatively infer that many of these 26S proteasome non-ATPase regulatory subunits may be associated with the ubiquitin-proteasome system and autophagy. They are at least involved in cell survival, proliferation, and metastasis processes, which might affect the occurrence of PAH. Besides, in our predictive analysis of potential therapeutic agents, we identified two proteasome inhibitors such as bortezomib and carfilzomib, as potential targets of these protein ubiquitination genes (Table 3). Recently, bortezomib was found to inhibit right ventricular systolic pressure (RVSP), right ventricular hypertrophy index (RVHI), and percent medial wall thickness (%MT) in MCT-induced pulmonary hypertension rats in an animal model of pulmonary arterial hypertension and to increase PTEN expression by inhibiting ubiquitination of PTEN protein, thereby inhibiting the PI3K/Akt pathway and improving pulmonary artery remodeling [74]. In addition, bortezomib has previously been shown to help treat pulmonary hypertension by interfering with intracellular calcium homeostasis in pulmonary artery smooth muscle cells (PASMC) and alleviating hypoxia-induced PASMC proliferation [75]. Altogether, we hypothesized that the ubiquitin-proteasome system might play an essential role in the occurrence of PAH, and some key mitochondria-related proteins may further mediate PAH development. Among the predicted results of targeted therapeutic agents, we also found that proteasome inhibitors may have a potential role in targeting dysregulated genes and thus also have potential applications for the treatment of PAH.

It is worth noting that our study has some drawbacks. In our study, a reagent-based method was used for mitochondrial isolation, which may cause the isolations to contain some cell membrane proteins. And due to the limited purity of the obtained mitochondrial fraction, we may not be able to draw more reliable conclusions without controlling the quality of purification. Generally speaking, we know that complete purification of mitochondria is still difficult, so this still needs to be explored to use new isolation methods to obtain higher purity mitochondria in tissues. Moreover, we noted three significant genes on downregulated proteins, including the identified Lipe, CAT, and Prkce. Although these genes were not the most significantly downregulated, they still maintained consistent significant downregulation in independent samples of rat lung tissue. Of concern is that clinical observational studies have found that the Cat gene is associated with pathophysiological processes in pulmonary vascular disease mediated by oxidative stress and is significantly downregulated in the development of emphysema, as well as in PAH and chronic thromboembolic pulmonary hypertension (CTEPH). At the same time, high expression of CAT is an essential factor in the prognosis of patients with pulmonary hypertension [76, 77]. Interestingly, the prediction result showed that the down mitochondrial-related genes such as Cat with the most regulations could be targeted and regulated by 26 miRNAs. In addition, we found that Prkce (named protein kinase C epsilon type), a calcium-independent, phospholipid- and diacylglycerol- (DAG-) dependent serine/threonine-protein kinase, also could play an essential role in the regulation of several cellular processes associated with cytoskeletal proteins and is involved in the regulation of immune response, cancer cell invasion, and apoptosis and was also shown lower expressed in rats with MCT-induced PAH [78]. Lipe (named hormone-sensitive lipase) was found with broad substrate specificity, catalyzing the hydrolysis of triacylglycerols, and could be the penultimate precursor of the pathway for de novo synthesis platelet-activating factor [79]. Nevertheless, all three downregulated genes showed low expression in studies reported on PAH. In contrast, all of them need to be further explored to investigate their specific molecular functions in the pathological mechanisms of PAH and consider their up- and downregulatory modalities further. Our study used the predicted miRNA target gene regulation results, and relevant experimental studies confirmed these regulatory relationships. Finally, these downregulated genes should also be considered as potential therapeutic targets to develop appropriate drugs to treat or reverse PAH. In the future, it should verify the expression of our identified consistent hub genes by Western blotting. In addition, clinical samples should be used to ascertain the role of hub proteins obtained from the animal model before using these results in clinical and therapeutic applications to treat PAH.

## 5. Conclusions

We have constructed a successful PAH model in rats by administrating MCT. Our study found 6 upregulated mitochondrial genes (*Psmd2*, *Psmc4*, *Psmc3*, *Psmc5*, *Psmd13*, and *Psmc2*) and 3 downregulated mitochondrial genes (*Lipe*, *Cat*, and *Prkce*) which are significantly differentially expressed in the lung tissue of PAH rats. First, Psmd2, Psmc4, Psmc3, Psmc5, Psmd13, and Psmc2 are upregulated in mitochondrial portions of PAH. Second, STRING-based analysis identified that these six genes are associated with mitochondrial functional enrichment. Third, we found that these six genes are consistently upregulated in an independent dataset. Fourth, our qPCR validation in rat liver tissue identified the consistent regulatory status of Psmd2, Psmc4, Psmc3, Psmc5, Psmd13, and Psmc2. Based on identifying potential targeting drugs, we noticed that two proteasome inhibitors, such as bortezomib and carfilzomib, are potential drugs targeting the deregulated genes, and these drugs should take more attention in the clinical trial of targeting the mitochondrial-associated hub genes in PAH. Altogether, mitochondrial-associated upregulated hub genes (*Psmd2*, *Psmc4*, *Psmc3*, *Psmc5*, *Psmd13*, and *Psmc2*) are substantial regulators during PAH development and progression.

## Figures and Tables

**Table 1:** The primers for qRT-PCR.

Primer	Sequence (5′–3′)
*Prkce* (rat)-F	GGCTGTCTTTCACGATGCTCCTATC
*Prkce* (rat)-R	TGGCTCCAGATCAATCCAGTCCTC
*Cat* (rat)-F	AGAAACCCACAAACTCACCTGAAGG
*Cat* (rat)-R	GAATCCCTCGGTCGCTGAACAAG
*Lipe* (rat)-F	AACCCGATTGTGGAAAGATGTCAGG
*Lipe* (rat)-R	GTGTGAGAATGCCGAGGCTGTATC
*Pmsd13* (rat)-F	GCCTGCCAACCACAGACAACTC
*Pmsd13* (rat)-R	ACCTCGTCTATGCTGCCTCTCAC
*Psmc3* (rat)-F	ACGAGGACTACATGGAGGGCATC
*Psmc3* (rat)-R	CGGACCAGCAGACAGACTAGAGG
*Psmc5* (rat)-F	GCTGTGGCTCATCATACGGACTG
*Psmc5* (rat)-R	TGCCCCTTCCCCAATGAATTTCTG
*Psmc4* (rat)-F	CATGATGCTCACCTCAGACCAGAAG
*Psmc4* (rat)-R	CGAAGTGCGTCAGTGGTAGTTCC
*Psmd2* (rat)-F	CTGCTCACCGTGCTTGTTTCTTTC
*Psmd2* (rat)-R	CTGGCAATGGTCGCAACTCCTC
*Gapdh* (rat)-F	AGAAGGCTGGGGCTCATTTG
*Gapdh* (rat)-R	AGGGGCCATCCACAGTCTTC

**Table 2:** The quantification of extracted proteins.

Group	OD value (595 nm)	Concentration (*μ*g/*μ*L)
Normal-1	1.092	1.90
Normal-2	1.005	1.56
Normal-3	0.890	1.11
Normal-4	0.959	1.38
Normal-5	1.050	1.73
Normal-6	0.998	1.53
MCT-1	1.014	0.80
MCT-2	0.925	1.24
MCT-3	1.311	2.76
MCT-4	1.082	1.86
MCT-5	0.965	1.40
MCT-6	0.829	0.497

**Table 3:** FDA-approved drugs potentially targeting hub mitochondrial genes.

Gene	Drug	Interaction types	Sources	PMIDs
*PSMD2*	Bortezomib	Inhibitor	DTC|MyCancerGenome|TdgClinicalTrial| ChemblInteractions|TEND	24524217
*PSMD2*	Carfilzomib	Inhibitor	DTC|MyCancerGenome|ChemblInteractions	24524217
*PSMC4*	Carfilzomib	Inhibitor	DTC|MyCancerGenome|ChemblInteractions	24524217
*PSMC4*	Bortezomib	Inhibitor	DTC|MyCancerGenome|ChemblInteractions	24524217
*PSMC3*	Carfilzomib	Inhibitor	DTC|MyCancerGenome|ChemblInteractions	24524217
*PSMC3*	Bortezomib	Inhibitor	DTC|MyCancerGenome|ChemblInteractions	24524217
*PSMC5*	Carfilzomib	Inhibitor	DTC|MyCancerGenome|ChemblInteractions	24524217
*PSMC5*	Bortezomib	Inhibitor	DTC|MyCancerGenome|ChemblInteractions	24524217
*PSMD13*	Bortezomib	Inhibitor	DTC|MyCancerGenome|ChemblInteractions	24524217
*PSMD13*	Carfilzomib	Inhibitor	DTC|MyCancerGenome|ChemblInteractions	24524217
*PSMC2*	Carfilzomib	Inhibitor	DTC|MyCancerGenome|ChemblInteractions	24524217
*PSMC2*	Bortezomib	Inhibitor	DTC|MyCancerGenome|ChemblInteractions	24524217

## Data Availability

The datasets used and analyzed during the present study are available from the corresponding author on reasonable request.

## Supplementary Materials

Supplementary Materials Supplementary Table S1: the label-free quantitative analysis of the mitochondrial proteome in the rat model. Supplementary Table S2: the significant mitochondrial DEPs in the PAH rat model when compared with control. Supplementary Table S3: identification of upregulated mitochondrial genes based on functional enrichment of the PPI network. Supplementary Table S4: identification of downregulated mitochondrial genes based on functional enrichment of the PPI network. Supplementary Figure S1: the visualization of the PPI network with upregulated mitochondrial DEPs. Supplementary Figure S2: the visualization of the PPI network with downregulated mitochondrial DEPs.
