# Supplementary figures and images for: Identifying Potential Mitochondrial Proteome Signatures Associated with the Pathogenesis of Pulmonary Arterial Hypertension in the Rat Model
Source: Oxid Med Cell Longev. 2022 Feb 21;2022:8401924. doi: 10.1155/2022/8401924 (PMC8885180; doi:10.1155/2022/8401924)

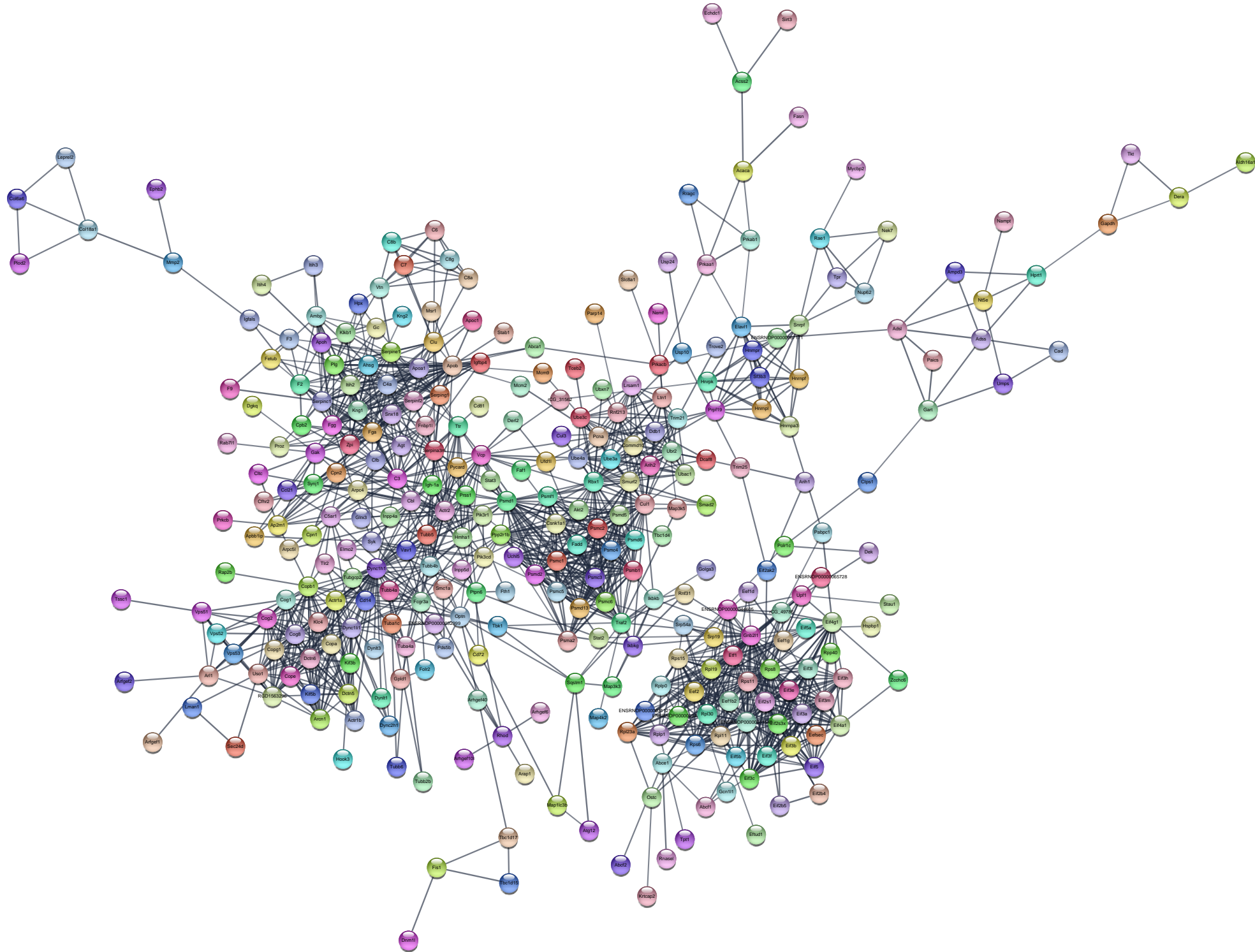

Supplement: Supplementary Materials — Supplementary Table S1: the label-free quantitative analysis of the mitochondrial proteome in the rat model. Supplementary Table S2: the significant mitochondrial DEPs in the PAH rat model when compared with control. Supplementary Table S3: identification of upregulated mitochondrial genes based on functional enrichment of the PPI network. Supplementary Table S4: identification of downregulated mitochondrial genes based on functional enrichment of the PPI network. Supplementary Figure S1: the visualization of the PPI network with upregulated mitochondrial DEPs. Supplementary Figure S2: the visualization of the PPI network with downregulated mitochondrial DEPs. [file 8401924.f1.zip › Supplementary Figure S1.pdf]

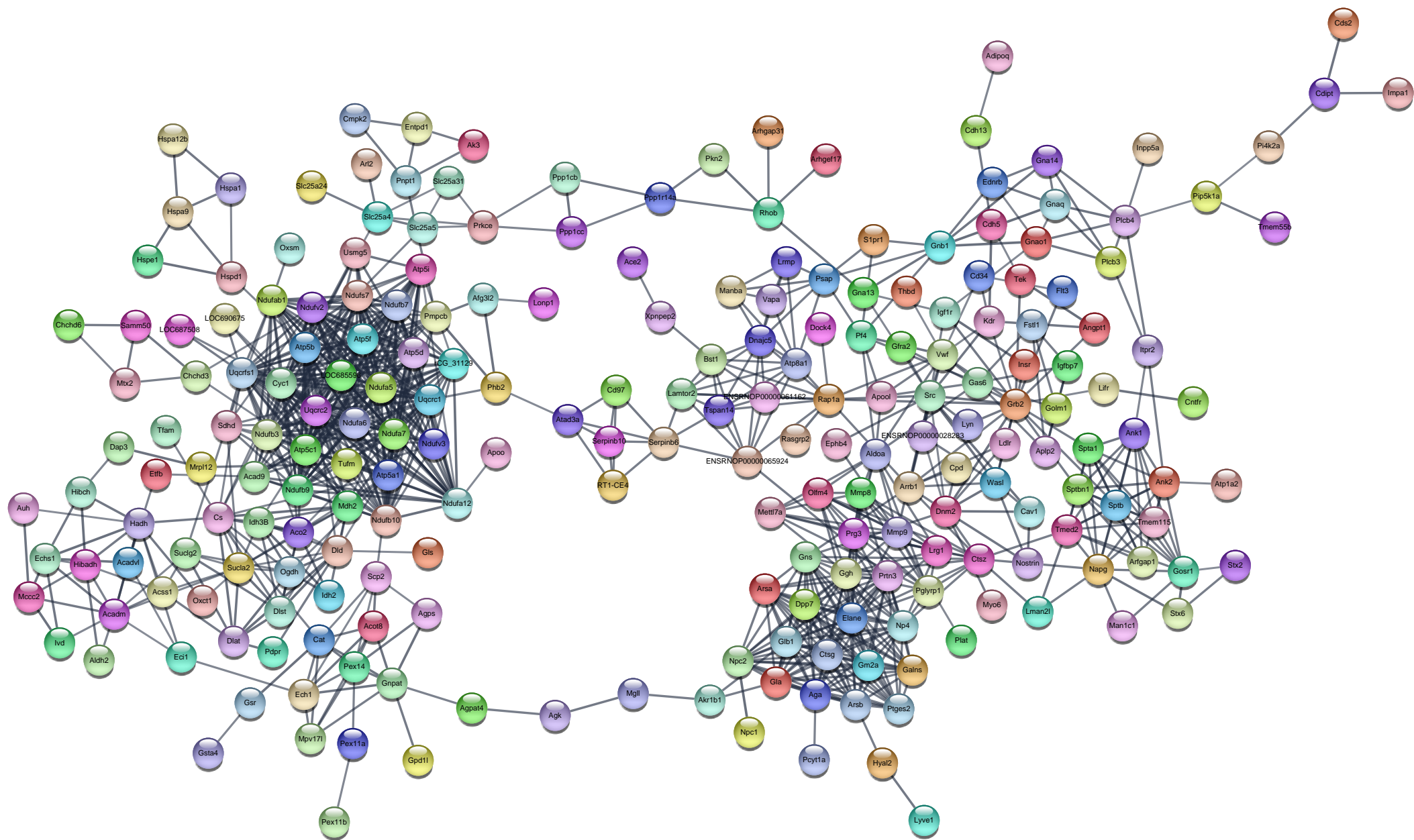

Supplement: Supplementary Materials — Supplementary Table S1: the label-free quantitative analysis of the mitochondrial proteome in the rat model. Supplementary Table S2: the significant mitochondrial DEPs in the PAH rat model when compared with control. Supplementary Table S3: identification of upregulated mitochondrial genes based on functional enrichment of the PPI network. Supplementary Table S4: identification of downregulated mitochondrial genes based on functional enrichment of the PPI network. Supplementary Figure S1: the visualization of the PPI network with upregulated mitochondrial DEPs. Supplementary Figure S2: the visualization of the PPI network with downregulated mitochondrial DEPs. [file 8401924.f1.zip › Supplementary Figure S2.pdf]
